# Efficacy and Safety of Epigallocatechin Gallate in the Treatment and Prevention of Dermatitis: A Systematic Review

**DOI:** 10.3390/biomedicines13061458

**Published:** 2025-06-13

**Authors:** Katarzyna Śladowska, Paweł Moćko, Tomasz Brzostek, Iwona Malinowska-Lipień, Michał Owca, Paweł Kawalec

**Affiliations:** 1 Department of Nutrition and Drug Research, Institute of Public Health, Faculty of Health Sciences, Jagiellonian University Medical College, Skawińska 8, 31-066 Kraków, Poland; katarzyna.wojcieszek@uj.edu.pl (K.Ś.);; 2 Health Policy and Management Department, Institute of Public Health, Faculty of Health Sciences, Jagiellonian University Medical College, Skawińska 8, 31-066 Kraków, Poland; pawel.mocko@uj.edu.pl; 3 Department of Internal Medicine and Geriatric Nursing, Institute of Nursing and Midwifery, Faculty of Health Sciences, Jagiellonian University Medical College, Kopernika 25, 31-501 Kraków, Poland; tomasz.brzostek@uj.edu.pl (T.B.); iwona.malinowska-lipien@uj.edu.pl (I.M.-L.)

**Keywords:** epigallocatechin gallate, dermatitis, efficacy, safety, systematic review

## Abstract

**Background/Objectives**: Epigallocatechin gallate (EGCG) is the most abundant catechin in green tea. Based on results from in vitro studies, EGCG—with its wide range of beneficial properties—has been considered a promising option for the treatment of patients with various skin conditions. **Methods**: The aim of this systematic review, conducted according to the Preferred Reporting Items for Systematic Reviews and Meta-Analyses (PRISMA) guidelines, was to evaluate the efficacy and safety of EGCG in the treatment and prevention of various types of dermatitis. **Results**: A search of PubMed, Embase, CENTRAL, and ClinicalTrials.gov databases identified eight trials (including four randomized trials) that investigated the use of EGCG alone (as a saline solution) for the treatment and prevention of radiation-induced dermatitis or as a component of a shampoo or cream for atopic and seborrheic scalp dermatitis. The identified single-arm and randomized controlled trials were characterized by low methodological quality, were in early phases of development, and/or included a small number of participants. The topical effect of EGCG on the severity of dermatitis was shown to depend on the type of dermatitis, concentration, and pharmaceutical form used. The administration of EGCG resulted in a significant reduction in skin symptoms in patients with radiation-induced dermatitis compared with placebo and with baseline, while in seborrheic dermatitis of the scalp, the results of shampoo with EGCG component were similar to those of active conventional treatment. The EGCG treatment was generally well tolerated, with no serious treatment-related adverse events. **Conclusions**: This study showed that EGCG can be a promising option for the treatment and prevention of various types of dermatitis. However, due to the small sample size, large, well-designed, randomized phase III trials are needed to confirm its safety and efficacy.

## 1. Introduction

Epigallocatechin gallate (EGCG), also known as epigallocatechin-3-gallate, is the ester of epigallocatechin and gallic acid. It is the most abundant catechin in green tea, *Camellia sinensis*, which belongs to the Theaceae family, accounting for approximately 50% of the total polyphenol content [1].

EGCG interacts with cell surface receptors, intracellular signaling pathways, and nuclear transcription factors, providing a wide range of antiangiogenic, anticancer, antioxidant, antimicrobial, and anti-inflammatory properties. These properties may be useful in the treatment of various neurological conditions, such as Parkinson’s and Alzheimer’s diseases, as well as cardiovascular, respiratory, and metabolic disorders [1,2,3,4]. The clinical application of EGCG is limited by its low bioavailability, as it is unstable in the alkaline environment of the intestinal tract and is easily oxidized in the circulatory system. Therefore, topical administration may optimize the therapeutic potential of EGCG by avoiding gastrointestinal digestion and minimizing the risk of adverse effects on other organs [5,6].

Dermatitis is a general term for conditions that cause inflammation of the skin, typically characterized by itchiness, redness, and a rash. There are various forms of dermatitis, each with its unique symptoms and causes. Numerous factors can lead to dermatitis, including exposure to irritating chemicals, excessive drying of the skin, infection with yeast such as *Malassezia furfur*, and high venous hydrostatic pressure. The most frequent types of dermatitis diagnosed by dermatologists are atopic dermatitis (AD) and contact dermatitis, while other types include irritant contact, allergic contact, seborrheic, and nummular dermatitis. These conditions commonly manifest as red rash, dry skin, and itchiness. Effective skin care can improve a patient’s quality of life and aid in the control of symptoms. For patients with mild symptoms, first-line treatment consists of topical therapies, including emollients and topical anti-inflammatory drugs. Emollients and moisturizers are essential for maintaining skin hydration and reinforcing the skin barrier. During flare-ups, topical corticosteroids are often prescribed to reduce inflammation and itching [7,8,9]. Despite the availability of numerous treatment options, patients receiving currently available topical therapies continue to experience reduced quality of life, sleep disorders, impaired overall functioning, and recurrent or inadequately controlled disease. Thus, the need for topical therapies that improve patient outcomes and disease control remains unmet [10,11].

Owing to its anti-inflammatory and antioxidant properties, EGCG may offer therapeutic benefits in the treatment of skin inflammation and damage. In vitro and in vivo studies in animal models indicate the potential utility of EGCG in the treatment of different types of dermatitis [12]. In a study using a mouse model of AD, Noh et al., 2008 demonstrated that the topical application of EGCG significantly reduced the total clinical severity score and ear thickness [13]. In addition, EGCG markedly decreased the levels of macrophage migration inhibitory factor and other cytokines associated with immune dysregulation, supporting its anti-inflammatory effects [13]. These findings were corroborated by Zhang et al., 2016 in a study on BALB/C mice with imiquimod-induced psoriasis-like dermatitis [14]. Topical application of EGCG attenuated the symptoms of psoriasiform dermatitis and improved pathological skin structure. In addition to its anti-inflammatory effects, EGCG also demonstrated immunomodulatory and antioxidant properties [14]. Furthermore, preclinical in vitro and in vivo evidence suggested that EGCG may inhibit radiation-induced damage [12]. Yi et al., 2020 investigated the radioprotective effects of EGCG in a model of oxidative damage induced by 60Coγ radiation in mice [15]. The results indicated that EGCG can serve as a natural radioprotector against radiation-induced damage.

Despite promising results from preclinical studies, comprehensive systematic reviews investigating the effectiveness of EGCG in the treatment of skin conditions in humans are lacking. Therefore, the aim of this systematic review was to evaluate the efficacy and safety of EGCG in the treatment of various types of dermatitis.

## 2. Methods

This systematic review was conducted according to the recommendations of the Preferred Reporting Items for Systematic Reviews and Meta-Analyses (PRISMA) Statement [16] and the Cochrane Handbook [17]. It was registered in the PROSPERO database (registration number CRD420251014134) [18].

The search strategy was conducted using Medical Subject Heading and Emtree terms related to the population and intervention, combined with Boolean logical operators, in Medline (via PubMed), Embase, and the Cochrane Central Register of Controlled Trials (CENTRAL) through 19 March 2025. The full search strategy was presented in detail in Supplementary Tables S1–S3. The ClinicalTrials.gov database was also searched for ongoing (unpublished) studies using the same criteria as for published trials (Supplementary Table S4).

A study was considered eligible if it met the following prespecified criteria: (1) inclusion of patients of any age diagnosed with any type of dermatitis; (2) assessment of EGCG use at any dose and via any route of administration; (3) design as a randomized controlled trial (RCT), a nonrandomized trial with a control group, or a single-arm study with at least 4 participants, published in English; and (4) assessment of any outcome related to the efficacy or safety of EGCG. Full-text articles and conference abstracts were included if they provided sufficient information regarding the study population, treatment regimen, and data necessary for extraction. Reviews, letters, editorials, in vitro experiments, and studies conducted on healthy volunteers or animals were excluded.

Two reviewers (K.Ś. and P.M.) independently conducted the search, using the same search strategy and study selection based on the previously established inclusion criteria. Studies were selected on the basis of the title and abstract and, if necessary, full-text articles. Any discrepancies were resolved by consensus with the third reviewer (P.K.). The degree of agreement between the reviewers reached 98%.

Data including study design (methodology), number of patients, patient characteristics, treatment regimen, treatment or follow-up duration, and results for the outcomes of interest were independently extracted from the selected studies by two reviewers (K.Ś., P.M.) using a predefined data extraction form. The quality of eligible RCTs was evaluated using the Cochrane Hanbook’s risk-of-bias tool 2.0 for RCTs [17,19]. The overall risk score was based on the highest level of risk identified in one of the assessed domains. The robvis tool [20] was used to graphically present the results of the risk of bias assessment for individual trials. The Newcastle–Ottawa Scale [21] was used to assess the quality of nonrandomized studies with a control group (cohort studies), while the National Institute for Health and Care Excellence (NICE) score was used for single-arm studies (case series).

After assessing the homogeneity of the studies, the feasibility of conducting a meta-analysis was evaluated. If a meta-analysis was not feasible, the results of each study were discussed separately according to the type of dermatitis evaluated.

## 3. Results

**Figure 1:**
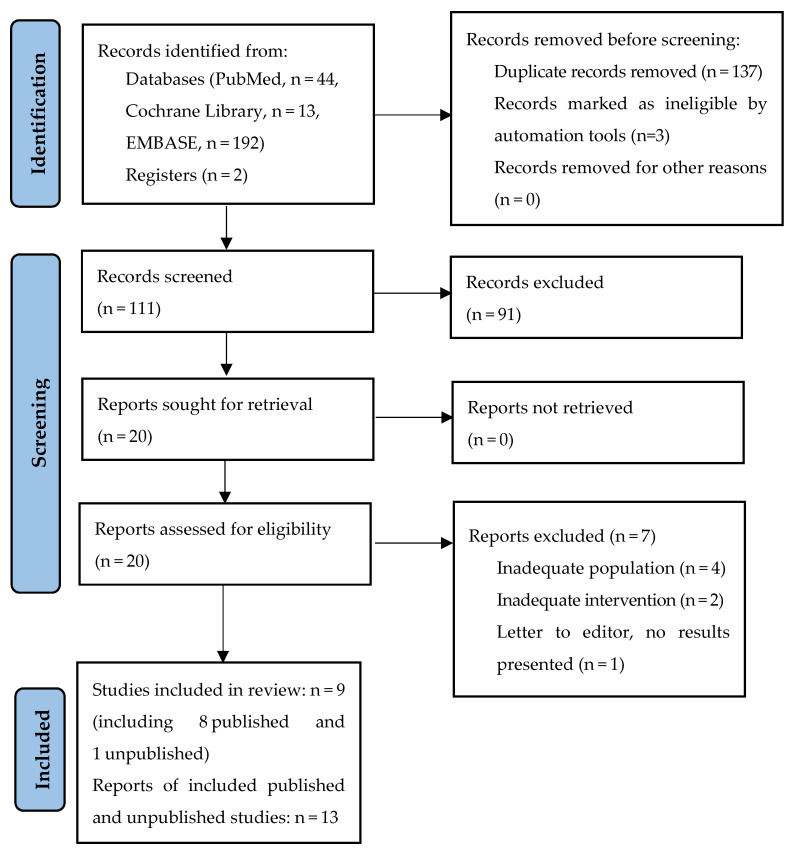
Search flow diagram.

The database search identified a total of 251 potentially relevant publications, of which 137 were duplicates, and three were excluded using automated tools. After screening the titles and abstracts of the remaining 111 records, 20 were considered eligible for full-text review. Of the 20 articles assessed in the full-text review, seven were excluded (four articles due to inadequate population, two due to inadequate intervention, and one due to no results presented), and the remaining 12 references, representing eight published studies, were included: Xie et al., 2023 [22], Zhao et al., 2022/Zhu et al., 2020 [23,24,25] (NCT02580279), Zhao et al., 2016 [26,27], Zhu et al., 2016/2015 [27,28,29], Zhu et al., 2023 [30], Patrizi et al., 2016 [31], Kim et al., 2014 [32], and Kim et al., 2019 [33]. Only one reference to an unpublished study was found: NCT04986384 [34] (Figure 1). The published studies included a total of 529 patients with different types of dermatitis. Most studies (N = 5) investigated the use of EGCG in radiation-induced dermatitis (Xie et al., 2023 [22], Zhao et al., 2022/Zhu et al., 2020 [23,24,25], Zhao et al., 2016 [26,27], Zhu et al., 2016/2015 [27,28,29], and Zhu et al., 2023 [30]). Two studies concerned scalp seborrheic dermatitis (Kim et al., 2014 [32], Kim et al., 2019 [33]), and one study was conducted in patients with atopic dermatitis (Patrizi et al., 2016 [31]). The methodology of the included studies is presented in detail in Table 1. The included studies varied considerably in terms of EGCG dosages and pharmaceutical formulations, study designs, treatment durations, and, in some cases, the severity of the dermatological conditions being treated. A meta-analysis was not performed due to substantial heterogeneity in study designs (randomized trials vs. single-arm trials), interventions (EGCG solution in 0.9% saline [22,23,24,25,26,27,28,29,30] or as a part of multi-component cream [31] or shampoo [32,33]), comparators (placebo vs. active therapies), populations, and outcome measures, as well as the limited number of studies available for each specific condition. Most included studies were early-phase or exploratory in nature, further limiting the feasibility and appropriateness of statistical pooling.

### 3.1. EGCG in the Treatment of Radiation-Induced Dermatitis

Three prospective single-arm phase I-I/II trials investigating the use of EGCG in the treatment of radiation-induced dermatitis were identified: Xie et al., 2023 [22], Zhao et al., 2016 [26,27], and Zhu et al., 2016/2015 [27,28,29]. These studies were conducted in small populations (21–49 patients) of patients with cancer in the chest area, primarily breast cancer. The NICE scores for all studies were 6 to 7 points (Supplementary Table S6). In all studies, a freshly prepared saline EGCG solution was sprayed on the affected area three times per day. The EGCG concentration varied between phase I trials. In the study by Xie et al., 2023 [22], high concentrations of 660 μmol/L, 1320 μmol/L, 1980 μmol/L, and 2574 μmol/L were used, but the treatment was started after the onset of grade III dermatitis. Zhao et al., 2016 [26,27] used lower concentrations: uptitrating from 40 µmol/L, 80 µmol/L, 140 µmol/L, 210 µmol/L, 300 µmol/L, and 440 µmol/L to 660 µmol/L, with EGCG administered to patients with grade I dermatitis. Based on the results reported by Zhao et al. in 2016 [26,27], an EGCG concentration of 660 µmol/L was selected for further evaluation in the phase I/II trial by Zhu et al. in 2016/2015 [27,28,29]. The treatment lasted 2 weeks in the study by Xie et al. in 2023 [22], whereas in the studies by Zhao et al. in 2016 [26,27] and Zhu et al. in 2016/2015 [27,28,29], EGCG was administered for 2 weeks after the end of radiation (generally around 4 weeks). In all trials, EGCG treatment led to a significant reduction (*p* < 0.05) in dermatitis symptoms such as pain, burning feeling, itching, tenderness, and pulling [22,26,27,28,29]. None of the patients required a delay in radiotherapy because of skin toxicity [22,26,27]. In the study by Xie et al., 2023 [22], EGCG demonstrated a relatively rapid effect, significantly reducing grade III radiation dermatitis to grade I or II within 3 days and 1 week of treatment (*p* < 0.001) (Table 2). Similarly, Zhao et al. in 2016 [26,27] and Zhu et al. in 2016/2015 [28,29] did not report the progression of dermatitis from grade I at baseline. In all trials, EGCG was well tolerated, and no treatment-related acute toxicity was reported. The only adverse event (AE) occurred in the study by Zhao et al. [26,27], who reported skin redness extending outside the radiation field immediately after EGCG administration in 4.2% of patients (Table 3).

### 3.2. EGCG in the Prevention of Radiation-Induced Dermatitis

The trial conducted by Zhao et al., 2022/Zhu et al., 2020 [23,24,25] assessed the use of EGCG in the prevention of radiation-induced dermatitis in women with breast cancer who received radiotherapy. In this double-blind, single-center phase II RCT, the risk of bias assessment raised some concerns (Supplementary Figure S1), mainly because not all patients were included in the efficacy analysis. The trial compared a freshly prepared EGCG solution (660 μmol/L) to placebo (0.9% saline solution). Both interventions were initiated from day 1 of radiotherapy, and the entire radiation field was sprayed three times per day throughout radiotherapy and for 2 weeks after its completion. Compared with placebo, EGCG treatment led to a significant reduction in the percentage of grade ≥ II dermatitis (*p* = 0.008), the mean Radiation-Induced Dermatitis Index (RIDI) score (*p* < 0.001), and the intensity of dermatitis-related symptoms, including itching (*p* < 0.001), tenderness (*p* = 0.002), and pain but not pulling (*p* = 0.27), based on the highest recorded RIDI score or symptom severity. Furthermore, the mean time to the onset of radiation-induced dermatitis was significantly longer in the EGCG-treated group compared with the placebo group (3.27 weeks vs. 2.89 weeks, *p* = 0.001), while a nonsignificant trend favoring EGCG was observed in the mean maximum increase in the difference in skin temperature (*p* = 0.10) (Table 2).

The majority of AEs recorded during the trial were related to anticancer treatment and included leukopenia, anemia, edema, fatigue, and radiation esophagitis, with no significant differences between the EGCG and placebo groups. Treatment-related AEs, including primarily local discomfort shortly after application, occurred in 3.6% and 3.7% of participants in the EGCG and placebo groups, respectively. No severe AEs related to EGCG treatment were noted in either group (Table 3).

In the study by Zhu et al., 2023 [30], some of the patients from the EGCG arm of the NCT02580279 trial [23,24,25] were matched with untreated control patients by age and cancer stage. The results confirmed the findings from the NCT02580279 trial [23,24,25], indicating that EGCG treatment can delay the onset of radiation-induced dermatitis compared with no EGCG use (*p* = 0.008) and substantially reduce the severity of dermatitis symptoms, as assessed by the RIDI and Radiation Therapy Oncology Group scores. Importantly, EGCG treatment did not affect survival outcomes, such as overall survival and disease-free survival [30].

### 3.3. EGCG in the Treatment of Atopic Dermatitis (AD)

Only one study assessing patients with AD was identified. This was a single-center study by Patrizi et al., 2016 [31] conducted in Italy. The risk of bias was assessed as high (Supplementary Figure S1), primarily due to concerns about allocation sequence generation. A total of 44 patients (29 women and 15 men; mean age, 22.6 years; range 6–69 years) were randomized at a 1:1 ratio to the placebo or MD2011001 groups. All participants had mild to moderate AD in the face and/or neck area. MD2011001 is a topical cream containing EGCG, vitamin E, and grape seed procyanidins (i.e., antioxidant and emollient ingredients thoroughly tested and widely employed in cosmetics). It was administered twice daily for 4 weeks in the areas affected by AD. Of the 44 patients, 39 were included in the analysis. During the study, six patients (one in the placebo group and five in the MD2011001 group) withdrew due to worsening of AD or AEs. Forty-three patients were included in the safety analysis [31].

No significant differences in the primary endpoint of the Investigator’s Global Assessment (IGA) score were reported between groups at any time point. A significant improvement in the mean IGA score from baseline was noted in each group; however, no significant differences were found between groups. A significant decrease in the lesional area in the skin on the face and neck was noted compared to baseline (day 28 vs. day 0) in both groups (Table 2). The reduction in the affected surface area was significant for the face in both groups and for the neck only in the MD2011001 group (day 7) [31].

The safety profiles in both groups were similar. Adverse events were observed in four patients in the placebo group and in seven patients in the MD2011001 group. The severity of AEs was usually mild or moderate, except for two severe cases in the MD2011001 group, which were unrelated to treatment in the opinion of the investigator. Irritant contact dermatitis occurred only in two cases (one in each group) and was considered uncertain in an additional two cases. No serious AEs were reported (Table 3) [31].

### 3.4. EGCG in the Treatment of Scalp Seborrheic Dermatitis

Two RCTs for scalp seborrheic dermatitis were identified—by Kim et al., 2014 [32] and Kim et al., 2019 [33]. Both studies were conducted at a single clinical center in Korea. The risk of bias was assessed as high for both studies (Supplementary Figure S1), primarily due to concerns about allocation sequence generation and concealment. Overall, 125 patients were randomized—75 patients in the study by Kim et al., 2014 [32] (mean age, 27 years; 27% males) and 50 patients in the study by Kim et al., 2019 [33] (mean age, 36 years; 30% males). In the study by Kim et al., 2014 [32], patients were randomly assigned to three treatment groups: new-formula shampoo (n = 25), 2% ketoconazole shampoo (n = 25), and 1% zinc-pyrithione shampoo (n = 25). In the study by Kim et al., 2019 [33], patients were randomized into two groups of 25 participants each: one group received a new-formula shampoo, and the other received a 1.5% ciclopirox olamine shampoo. In both studies, EGCG was one of the components of the new-formula shampoo, along with *Rosa centifolia* petals, zinc pyrithione, and climbazole. The assessments were performed at baseline and after 2 and 4 weeks. A total of 72 participants completed the study by Kim et al., 2014 [32] (one patient in the new-formula shampoo group did not use the product properly, and two patients in the zinc-pyrithione shampoo group withdrew due to personal reasons). In the study by Kim et al., 2019 [33], two patients were lost to follow-up: one due to a protocol violation (application of topical corticosteroid to the scalp) and the other due to unspecified AE.

The results of both studies indicated comparable effectiveness of the assessed interventions. In the study by Kim et al., 2014 [32], the clinical severity score improved significantly at 2 and 4 weeks vs. baseline in all groups, but no differences were noted between groups. No changes in sebum secretion were observed, either between groups or relative to baseline. Significant differences in sebum secretion were found only for scalp location, with the highest secretion in the frontal area and the lowest in the temporal area (Table 2) [32].

Kim et al., 2019 [33] also reported a significant improvement in the clinical severity score at weeks 2 and 4 vs. baseline in both groups. The mean changes in the clinical severity scores at weeks 2 and 4 did not show significant differences between groups. In addition, sebum secretion levels decreased in both groups at weeks 2 and 4 compared with baseline [33].

In summary, both studies indicated the potential benefits of EGCG for improving clinical severity scores and sebum secretion. However, the new-formula shampoo was comparable with other interventions.

### 3.5. Unpublished Study

The only unpublished study [34] evaluating the use of EGCG in dermatitis was a randomized, open-label, crossover study investigating the topical application of a new spray composed of ambora extract and EGCG combined with enoxolone in moderate-to-severe childhood eczema. The study compared two groups: one receiving immediate treatment with the spray and another receiving delayed treatment. As the trial has already been completed, the publication of the results can be expected soon.

## 4. Discussion

The aim of this systematic review was to determine the efficacy and safety of EGCG for the treatment of different types of dermatitis based on the available data. In contrast to previous reviews, we conducted a systematic review using a rigorous methodology based on the PRISMA Statement [16] and the Cochrane Handbook [17]. Data collection and extraction were performed independently by two authors. Finally, our review comprehensively evaluated key clinical efficacy and safety endpoints related to dermatitis treatment. In addition, the risk of bias and credibility of each study were assessed using appropriate dedicated scales.

Despite the growing number of studies on EGCG, data on its clinical efficacy and safety for patients with dermatitis are limited. The identified studies differed in terms of the indication for EGCG use, its dose and pharmaceutical form, and the treatment period.

Of the eight published studies, five addressed the use of EGCG in the treatment or prevention of radiation-induced dermatitis [22,23,24,25,26,27,28,29,30]. Apart from one randomized placebo-controlled phase I/II study [23,24,25], the remaining studies were primarily single-arm and early-phase I studies. As such, they were limited to assessing EGCG effects relative to baseline values before treatment initiation. Nevertheless, the results suggested that saline EGCG solution is effective in the prevention and treatment of radiation-induced dermatitis by reducing symptoms such as pain, burning sensation, itching, tenderness, and pulling as compared to placebo (saline) and baseline values.

In the context of acute radiation-induced dermatitis, the treatment duration used in the included studies appears appropriate, as EGCG is typically administered during and shortly after the cessation of the triggering factor—namely, radiotherapy. This timing aligns with the natural course of the condition and the therapeutic window for intervention [35].

Importantly, the topical use of EGCG does not affect survival rates in radiation-induced dermatitis. These findings are promising, especially considering the favorable safety profile. They are also consistent with preclinical studies, which showed that EGCG increases human skin cell viability and reduces X-ray–induced apoptosis [36]. By binding to free radicals, intercalating into DNA, and repairing the damage caused by free radicals, EGCG protects deoxyribonucleic acid from radiation-induced damage. Moreover, by blocking the activity of the proteasome, a crucial regulator of inflammation, EGCG can inhibit the production of proinflammatory cytokines such as tumor necrosis factor-α or interleukins IL-1β, IL-6, and IL-8 [37].

From a clinical perspective, the need to freshly prepare EGCG solutions each time may be burdensome for patients and medical personnel due to the limited stability of EGCG in water solutions. The identified studies did not address the convenience of using the EGCG solution or whether it remains in place after application, which is an important consideration given that it is a saline solution rather than a cream or ointment. From a pharmacological perspective, EGCG’s instability and limited bioavailability—particularly in topical applications—remain important challenges. However, several of the included studies have already addressed these limitations by testing various formulation strategies, such as creams and shampoo, aimed at improving compound stability, skin penetration, and patient tolerability. This diversity in pharmaceutical approaches represents a notable strength of the current body of evidence and reflects ongoing efforts to optimize EGCG delivery in clinical settings. While EGCG is only sparingly soluble in pure water and some water solutions [38,39], the EGCG concentrations in 0.9% saline solution were sufficient to produce a clinical effect in studies in the population with radiation-induced dermatitis.

Different results were obtained by Patrizi et al., 2016 in a small RCT including patients with mild to moderate AD localized on the face and neck [31]. The treatment group received MD2011001 cream containing EGCG, vitamin E, and grape seed procyanidin; therefore, it is difficult to determine which component was primarily responsible for its effects, and the control group received a cream without these active ingredients. A significant improvement from baseline was found in the intensity of dermatitis symptoms such as erythema, papules, excoriation, and xerosis, along with a progressive reduction in the extent of the affected area both in the treatment and control groups. No significant differences were shown between groups. Only a trend favoring the EGCG-containing cream was observed, but significantly fewer patients in the treatment group reported symptoms at the site of application as compared to those who applied the placebo cream. The lack of significant differences may be attributed to the small sample size and short study duration (up to 28 days), given the variable and chronic course of AD characterized by periods of exacerbations and remissions. Another possible reason is a minor difference in baseline patient characteristics, including a slightly higher AD severity in the study group compared with controls, particularly regarding the IGA score and the extent of the lesional area. Furthermore, the study did not specify the concentration of EGCG or other ingredients used in MD2011001, making it difficult to compare it to other methods of local EGCG administration in different types of skin inflammation.

In two small RCTs, Kim et al., 2014 [32] and Kim et al., 2019 [33] compared a new formula shampoo containing EGCG, *Rosa centifolia* petals, zinc pyrithione, and climbazole to conventional treatment options such as ketoconazole, zinc-pyrithione shampoos, and ciclopirox olamine for the treatment of seborrheic dermatitis. The effectiveness of the shampoo containing 0.005% EGCG was comparable to standard options, suggesting that the tested shampoo may serve as an alternative to these therapies. The goal of treatment in seborrheic dermatitis is to reduce sebum production and to provide anti-inflammatory, antimicrobial, and antioxidant effects. The precise cause of scalp seborrheic dermatitis, a chronic form of inflammatory dermatosis, is unknown, but it is associated with sebum production and the growth of *Malassezia* fungus. However, as the tested shampoo contained other active ingredients in addition to EGCG, it is difficult to determine which component was primarily responsible for its effects. For example, zinc pyrithione and climbazole are preservatives with antifungal properties [40,41]. Nevertheless, EGCG is known for its antisebum, anti-inflammatory, and antioxidative effects [42]. By inhibiting the nuclear factor κB and AP-1 pathways and modifying the AMP-activated protein kinase–sterol regulatory element-binding protein-1 signaling pathway, EGCG decreased sebum and inflammation in SEB-1 sebocytes [43]. Additionally, EGCG was shown to promote hair growth [44]. After 4 weeks of treatment, IL-10 levels in patients receiving an EGCG-containing shampoo significantly increased. Patients who had higher baseline IL-10 levels showed a better response to shampoo treatment. Since IL-10 is a well-known cytokine that reduces inflammation, it was suggested that the new shampoo with EGCG would help control IL-10 production [32]. However, despite promising results, the 4-week treatment period in both studies seems to be relatively short, given the chronic nature of seborrheic dermatitis and the need for long-term use of the product [32,33].

Despite strict methodology, this review has several limitations, including the small number of trials conducted in populations with AD, psoriasis, seborrheic dermatitis, and eczema, as well as a relatively short follow-up duration (up to 4 weeks). Such a short follow-up may be insufficient to evaluate the duration of EGCG treatment effects in chronic conditions such as AD or seborrheic dermatitis. Another limitation is the lack of sufficient data from studies directly comparing EGCG with other active regimens in patients with radiation-induced dermatitis. Moreover, the identified single-arm and randomized controlled trials are generally of low methodological quality (i.e., with unclear or high risk of bias), are in early phases of development, and often include small sample sizes, which limits the generalizability and robustness of the findings. Considering these limitations and the differences between the included studies, our results should be interpreted with caution. Interestingly, there is little ongoing research [45] on the use of EGCG in the treatment of dermatitis despite intensive exploration of its use in other conditions, such as cancer, Alzheimer’s disease, and multiple sclerosis.

## 5. Conclusions and Future Direction

Available scientific evidence from small single-arm and small RCTs with unclear or high risk of bias shows promising results for the topical use of EGCG in the treatment of dermatitis, such as radiation-induced dermatitis and scalp seborrheic dermatitis. However, given the limitations of the available studies, further research is needed to evaluate the effects of EGCG in patients with various types of dermatitis. In particular, RCTs with active comparators, larger patient populations, and long-term follow-up are necessary for generating more robust evidence.

## Figures and Tables

**Table 1:** Methodology of included published studies regarding the use of EGCG in the treatment or prevention of dermatitis.

Trial	Methodology	Population	Assessed Dose of EGCG and Administration Route	Length of Treatment/Follow-Up
**Radiation-induced dermatitis—treatment and prevention**				
Xie et al., 2023 [22]	Phase I, prospective, two-center, single-arm	Adult patients with breast cancer, lung cancer, or esophageal cancer received definitive radiation therapy in which the target included supraclavicular area, with acute grade III radiation-induced dermatitis (N = 21, data analyzed from 19 patients)	EGCG (freshly diluted in 0.9% saline) at concentration: 660 μmol/L, 1320 μmol/L, 1980 μmol/L, 2574 μmol/L. The solution was sprayed 3 times a day at 0.05 mL beyond the whole radiation field	2 weeks (15 days)
Zhao et al., 2022/Zhu et al., 2020 [23,24,25] (NCT02580279)	Phase II, randomized (2:1), double-blind, single-center	Adult women with breast cancer, 2 to 3 weeks after the completion of adjuvant chemotherapy and who received radiotherapy (N = 180 enrolled, 165 patients evaluable)	**Prevention:** EGCG (freshly prepared solution (660 μmol/L) vs. placebo (0.9% saline solution) Both interventions initiated from day 1 of radiotherapy until 2 weeks after radiotherapy completion and sprayed on the whole radiation field using a sterilized medical sprayer, 3 times a day at 0.05 mL/cm^2^	From first day of radiotherapy until 2 weeks after radiotherapy completion
Zhao et al., 2016 (part of NCT01481818) [26,27]	Phase I, prospective, single-arm	Adult women with breast cancer with a planned course of radiotherapy to the chest wall after modified radical mastectomy (N = 24)	EGCG (freshly diluted in 0.9% saline) at concentrations escalated from 40 µmol/L, 80 µmol/L, 140 µmol/L, 210 µmol/L, 300 µmol/ and 440 µmol/L to 660 µmol/L. The solution was sprayed 3 times a day at 0.05 mL/cm^2^ to 2 cm beyond the whole radiation field. Three patients were assigned to each dose level. If no dose-limiting toxicity (grade > 1) was observed, the next level was opened	EGCG administration was initiated once grade I dermatitis occurred and continued until 2 weeks after the end of radiation (median duration of EGCG treatment: 4 weeks)
Zhu et al., 2016/2015 (part of NCT01481818) [27,28,29]	Phase I/II, prospective, single-arm	Adult women with breast cancer with a planned course of radiotherapy to the chest wall after modified radical mastectomy (N = 49)	EGCG (freshly diluted in 0.9% saline) at concentration 660 µmol/L. The solution was sprayed 3 times a day at 0.05 mL/cm^2^ at the whole radiation field.	EGCG administration was initiated once grade I dermatitis occurred and continued until 2 weeks after the end of radiation (mean duration of EGCG treatment: 4 weeks)
Zhu et al., 2023 [30]	Cohort study (patients treated with EGCG in phase II trial NCT02580279 matched with control patients not treated by EGCG; stage-and age-matched, selected at random from the medical database of the hospital)	Women with stage III breast cancer, who undergo radiotherapy (N = 86)	**Prevention:** EGCG (no precise data about dose but similar as in phase II NCT0258027 trial) vs. no EGCG treatment	No information about duration of EGCG treatment, (probably similar as in phase II NCT02580279 trial). Follow-up—up to 4 years (median 50.6 months in EGCG group and median 48.6 months in no EGCG group)
**Atopic dermatitis**				
Patrizi et al., 2016 [31]	Randomized (1:1), double-blind, single-center	Patients aged ≥ 6 years with mild-to-moderate AD with a cephalic distribution and a particular involvement of the areas around the mouth and/or the eyelids/periocular area and/or the neck, with IGA score 2 or 3 (N = 44; 39 included in efficacy and 43 in safety analysis)	MD2011001—nonsteroidal topical cream containing vitamin E, EGCG and grape seed procyanidins vs. placebo (soothing cream containing the same ingredients as the MD2011001 cream, with the exclusion of vitamin E, EGCG and grape seed procyanidins) Both study products were applied twice daily on the affected areas of the face and/or neck	Up to 28 days
**Scalp seborrheic dermatitis**				
Kim et al., 2014 [32]	Randomized (1:1:1), double-blind, single-center	Patients with scalp seborrheic dermatitis with a clinical severity score of ≥3 with faint pink or more severe erythema, and scraped or more severe dandruff (N = 75)	New-formula shampoo (0.01% extract *of Rosa centifolia* petals, 0.005% EGCG, 0.3% zinc pyrithione and 0.45% climbazole) vs. 2% ketoconazole shampoo (Nizoral) vs. 1% zinc-pyrithione shampoo (Head & Shoulder) Patients were instructed to massage their scalps for at least 5 min with the assigned shampoo and then rinse with water three times a week	4 weeks
Kim et al., 2019 [33]	Randomized (1:1), probably open-label, single-center	Patients with scalp seborrheic dermatitis with a clinical severity score of >3 (N = 50, 48 patients included in efficacy analysis)	New-formula shampoo (0.01% extract of *Rosa centifolia* petals, 0.005% EGCG, 0.3% zinc pyrithione and 0.45% climbazole vs. 1.5% ciclopirox olamine shampoo Patients were instructed to massage the assigned shampoo onto their scalps for at least 5 min and then rinse it off with water thrice a week	4 weeks

AD—atopic dermatitis; IGA—Investigator’s Global Assessment, based on a six-point scale (0—clear, 1—almost clear, 2—mild, 3—moderate, 4—severe, 5—very severe); EGCG—epigallocatechin gallate.

**Table 2:** Efficacy outcomes of EGCG from included studies.

Trial	Trial Arms	N	Method of Assessing the Severity of Skin Lesions and Other Important Outcomes	Assessment of the General Severity of Skin Lesions as a Result of EGCG Use	Assessment of Individual Symptoms of Dermatitis	Other Endpoints
**Radiation-induced dermatitis—treatment and prevention**						
Xie et al., 2023 [22]	EGCG	19	- RTOG score for assessment of grade of dermatitis; - STAT for assessment-related dermatitis symptoms included erythema, burning feeling, itching, pulling, and pain.	All grade III radiation dermatitis significantly decreased to grade I or grade II after 3 days and 1 week of EGCG use (*p* < 0.001) After 15 days of EGCG treatment: - 17/19 (89.5%) patients with no dermatitis; - 1/19 (5.25%) patients with grade I dermatitis; - 1/19 (5.25%) patients with grade I dermatitis.	At the last follow-up of EGCG treatment, significant relief vs. baseline in: - Burning sensation (*p* < 0.001); - Tractive sensation (*p* < 0.001); - Tenderness (*p* < 0.001); - Erythema (*p* < 0.001); - Itching (*p* < 0.001); - Pain (*p* < 0.001).	No radiation therapy delay or interruption
Zhao et al., 2022/Zhu et al., 2020 [23,24,25] (NCT02580279)	EGCG	111	- RTOG score for assessment of grade of dermatitis; - STAT for assessment-related dermatitis symptoms included erythema, burning feeling, itching, pulling, and pain; - RIDI calculation method was used for the 5 RID-related symptom index; - Time to the onset of dermatitis; - Body temperature.	EGCG vs. placebo: - Incidence of ≥2 dermatitis: 50.5% vs. 72.2%, *p* = 0.008; - Incidence of ≥3 dermatitis: 3.6% vs. 9.3%, *p* = 0.16; - Mean RIDI (SD): 5.22 (1.60) vs. 6.21 (1.56), *p* < 0.001.	EGCG vs. placebo (taking into account the highest score or RID and symptom): - Itching: *p* < 0.001 (favoring EGCG); - Pulling: *p* = 0.27 (favoring EGCG); - Pain: *p* = 0.03 (favoring EGCG); - Tenderness: *p* = 0.002 (favoring EGCG). EGCG vs. placebo (score ≥2 event rate); HR [95% CI]: - Pain: HR = 0.41 [0.18; 0.91], *p* = 0.03; - Burning feeling: HR = 0.31 [0.15; 0.63], *p* = 0.001; - Itching: HR = 0.24 [0.12; 0.50], *p* < 0.001; - Pulling: HR = 0.48 [0.19; 1.22], *p* = 0.12; - Tenderness: HR = 0.32 [0.16; 0.64], *p* = 0.001.	EGCG vs. placebo: - mean (SD) appearance time of RID: 3.27 [0.86] weeks vs. 2.89 [0.60] weeks, *p* = 0.001; - mean (SD) maximum increase in the difference in skin temperature: 1.18 (0.73) vs. 1.51 (0.99); *p* = 0.10.
	Placebo (saline)	54				
Zhao et al., 2016 (NCT01481818) [26,27]	EGCG	24	- RTOG score for assessment of grade of dermatitis; - STAT for assessment-related dermatitis symptoms included erythema, burning feeling, itching, pulling, and pain	Grade II dermatitis developed from Grade I at the end of radiotherapy in 16.7% of patients. 16.7% more patients with Grade II dermatitis were found at 1 week after the radiotherapy. As the EGCG treatment was performed continuously, all these Grade II reactions were decreased to Grade I at 2 weeks after the end of radiotherapy.	After 1 week of EGCG treatment of significant relief vs. baseline in: - Itching (*p* < 0.001); - Tenderness (*p* < 0.001); - Pain (*p* < 0.001); - Burning (*p* < 0.001); - Pulling (*p* < 0.032). 2 weeks after the end of radiotherapy vs. beginning of the EGCG treatment, relief in: - Itching (*p* < 0.001); - Tenderness (*p* < 0.001); - Pain (*p* < 0.001); - Burning (*p* < 0.001); - Pulling (*p* < 0.840).	No patient needed delay in radiotherapy because of skin toxicity.
Zhu et al., 2016/2015 (part of NCT01481818) [27,28,29]	EGCG	49	- RTOG score for assessment of grade of dermatitis; - STAT for assessment-related dermatitis symptoms included erythema, burning feeling, itching, pulling, and pain	Maximum radiation-induced skin toxicity observed during EGCG treatment was as follows: - grade I: 71.4%; - grade II: 28.6%; - grade III-IV: 0%. Significant difference between the onset and the end of the study in RTOG scores (N = 49, *p* < 0.05). RTOG score was not increased during radiotherapy in 71.4% of patients	STAT score before vs. within 1 week of EGCG therapy—significant relief in: - Pain (*p* < 0.05); - Burning-feeling (*p* < 0.05); - Itching (*p* < 0.05); - Tenderness (*p* < 0.05); - Pulling (*p* = 0.035). STAT score before vs. end of study—significant relief in: - Pain (*p* = 0.004); - Burning-feeling (*p* < 0.05) - Itching (*p* < 0.05); - tenderness (p = 0.008).	EGCG can significantly and persistently control the symptoms of: - pain (85.7% of patients); - burning-feeling (89.8%); - itching (87.8%); - pulling (71.4%); - tenderness (79.6%)
Zhu et al., 2023 [30]	EGCG	43	- RIDI; - RTOG score; - Time to the onset of dermatitis; - Survival rates.	RIDI in the EGCG group vs. no EGCG group (2.56 vs. 3.36; *p* = 0.002) Better RTOG score for the EGCG group during radiotherapy vs. no EGCG group (*p* = 0.003)	-	Dermatitis in the EGCG group appeared later than it did in no EGCG group (3.19 weeks vs. 2.67 weeks; *p* = 0.008). No significant difference in overall survival, disease-free survival and freedom from locoregional and distant failure between groups (*p* > 0.05)
	No EGCG	43				
**Atopic dermatitis**						
Patrizi et al., 2016 [31]	Cream with EGCG, vitamin E and grape seed procyanidins)	20	- Severity was assessed using IGA (therapeutic success defined as IGA 0 or 1; treatment failure defined as IGA > 2); - Change in the surface of the lesional area and the variation in the severity of subjective symptoms (itching and/or burning); - Severity of six individual signs (erythema, papules/edema, vesiculation/crusting, excoriation, lichenification/prurigo and xerosis) was evaluated using a four-point scale (0—absent, 1—mild, 2—moderate, 3—severe). The same four-point scale was utilized by patients to report the severity of subjective symptoms; - PGA.	Cream with EGCG vs. placebo at 7 days of treatment: - Patients with therapeutic success: 70% vs. 79%, *p* > 0.05. Cream with EGCG vs. placebo at 28 days of treatment: - Patients with therapeutic success: 70% vs. 89%, *p* > 0.05. Statistically significant reduction of IGA score during the treatment period as compared to the baseline in each group.	Cream with EGCG vs. placebo at 28 days of treatment—severity of signs and symptoms (mean values referred to the total face and neck area (severity score based on a four-point scale): - Erythema: 0.7 vs. 0.04 (*p* > 0.05); - Papules: 0.2 vs. 0.05 (*p* > 0.05); - Vesicles/crusts: 0.3 vs. 0.05; - Escoriation: 0.4 vs. 0.05 (*p* > 0.05); - Lichenification: 0.6 vs. 0.2 (*p* > 0.05); - Xerosis: 0.4 vs. 0.4 (*p* > 0.05); - Symptoms: 0.4 vs. 0.2 (*p* > 0.05). Statistically significant reduction of the above signs and symptoms at day 28 as compared to the baseline in each group, except vesicles/crusts	Lesion area: - Statistically significant reduction in lesion area at 28 days as compared to the baseline in each group; - No significant difference at day 28 between groups (*p* > 0.05). Cream with EGCG vs. placebo: - Rescue therapy: 20% vs. 15.8%; - Symptoms reported (defined as itching, burning or discomfort) at the site of the application of the study product: 24% vs. 49%, *p* = 0.034. Approximately 90% of patients considered the tolerability of the study treatment good or excellent, and nearly half perceived the study products as better tolerated than topical preparations used in the past.
	Placebo (cream without EGCG, vitamin E and grape seed procyanidins)	19				
**Scalp seborrheic dermatitis**						
Kim et al., 2014 [32]	shampoo (Rosa centifolia petals, EGCG, zinc pyrithione, climbazole	25	- Clinical severity score—clinical features of erythema, dandruff, and lesion extent were measured according to their severity on a four-point scale (0–3, where 0 is no signs and 3—the most severe signs); - Subjective improvement assessed on a five-point scale (1, much better; 2, somewhat better; 3, no change; 4, somewhat worse; and 5, much worse); - User satisfaction—foam richness and hair smoothness while rinsing and after drying on a five-point scale (1, excellent; 2, good; 3, moderate; 4, poor; and 5, very bad).	Clinical severity score improved significantly relative to baseline at weeks 2 and 4 in all groups (*p* < 0.05). The changes in clinical severity score at weeks 2 and 4 did not differ significantly between the three groups (*p* = 0.39 and 0.63, respectively)	The changes in clinical severity subscores (i.e., for erythema, dandruff, and lesion extent) at weeks 2 and 4 did not significantly differ between the three groups (*p* = 0.55, 0.53, and 0.18, respectively, at week 2; and *p* = 0.68, 0.57, and 0.83 at week 4).	Patients’ subjective improvement scores did not differ significantly between the three groups at weeks 2 (*p* = 0.17) and 4 (*p* = 0.83) Changes in sebum secretion did not differ significantly between the three groups at weeks 2 and 4 (*p* = 0.29 and 0.53, respectively); no significant changes were noted in the three groups as compared with baseline. Patients’ subjective improvement scores did not differ significantly between the three groups at weeks 2 (*p* = 0.17) and 4 (*p* = 0.83) Foam richness was superior in the new-formula group compared with the ketoconazole group (*p* = 0.013), and smoothness while rinsing was superior in the zinc pyrithione group compared with the ketoconazole group (*p* = 0.011).
	ketoconazole shampoo	25				
	zinc-pyrithione shampoo	25				
Kim et al., 2019 [33]	shampoo (Rosa centifolia petals, EGCG, zinc pyrithione, climbazole)	25	- Clinical severity score—clinical features of erythema, dandruff, and lesion extent—four-point scale of severity (0–3, where 0 is no signs and 3—the most severe signs); - Patients’ improvement scores—five-point scale (5—much better; 4—somewhat better; 3—no change; 2—somewhat worse; 1—much worse); - User satisfaction—five-point scale; the patients assessed foam richness and hair smoothness while rinsing off the shampoo and after drying their hair (5—excellent; 4—good; 3—moderate; 2—poor; 1—very bad); - Sebum secretion.	Clinical severity score improved significantly vs. baseline at weeks 2 and 4 in both groups (*p* < 0.01) The changes in clinical severity score at weeks 2 and 4 did not differ significantly between the groups (*p* = 0.63)	-	A significantly greater number of patients responded “much better” in the new-formula shampoo-treated group (56.0% vs. 21.7%; *p* < 0.05). Foam richness, hair smoothness while rinsing off the shampoo, and hair smoothness after drying were significantly higher in the new-formula shampoo-treated group (all *p* < 0.05) Changes in sebum secretion did not differ significantly between the groups at 4 weeks (*p* = 0.39); significant changes were noted in both groups as compared to baseline at 2 and 4 weeks (*p* < 0.01).
	ciclopirox olamine shampoo	23			-	

IGA—Investigator’s Global Assessment, based on a six-point scale (0—clear, 1—almost clear, 2—mild, 3—moderate, 4—severe, 5—very severe). EGCG—epigallocatechin gallate. PGA—Patient’s Global Assessment, based on a six-point scale (0—clear, 1 -almost clear, 2—mild, 3—moderate, 4—severe, 5—very severe). POEM—Patient Orientated Eczema Measure. RIDI—Radiation-Induced Dermatitis Index. RTOG—Radiation Therapy Oncology Group with Grade description: 0—no change over baseline, 1—follicular, faint or dull erythema/epilation/dry desquamation/decreased sweating, 2—tender or bright erythema, patchy moist desquamation/moderate edema, 3—confluent, moist desquamation other than skin folds, pitting edema, 4—ulceration, hemorrhage, necrosis. Grade 0 is no radiation-induced dermatitis. Grade 1 is considered a mild score. Grade 2 is considered a moderate score, and Grade 3–4 is considered a severe score. STAT—Skin Toxicity Assessment Tool; the STAT scores range from 0, representing no symptoms, to 5, representing the worst symptoms. TEWL—transepidermal water loss.

**Table 3:** Safety outcomes from included studies.

Trial	Trial Arms	AEs (%)	Serious or Severe AEs (%)	5 Most Common AE	Dose Reduction Due to AEs (%)	Discontinuation Due to AEs (%)	Comment on the Safety Assessment from Reference
Xie et al., 2023 [22]	All patients, N = 19	0%, including 0% related AEs	Serious: 0%	-	0%	0%	EGCG was well tolerated by all patients. The highest dose of this phase I trial (2574 μmol/L) was recommended for continuous Phase II trial for further evaluation.
Zhao et al., 2022/ Zhu et al., 2020 [23,24,25] (NCT02580279)	EGCG	3.6% related to treatment	Severe: 0% related to the treatment	EGCG vs. placebo (mostly related to cancer and/or treatment—not related with EGCG or placebo): - Leukopenia (53.2% vs. 57.4%), *p* = 0.61; - Upper limb oedema: 32.4% vs. 25.9%, *p* = 0.39; - Anemia: 19.8% vs. 20.4%, *p* = 0.93; - Radiation esophagitis: 18.9% vs. 18.5%, *p* = 0.95; - Fatigue: 16.2% vs. 18.5%, *p* = 0.71. Related to EGCG or placebo: - Overall 3.6%—discomfort within 10 min after drug application, considered to be related to radiotherapy and local drug application (grade 1 and grade 2 pricking skin sensation); - 0.9% treated with EGCG with grade I pruritus.	-	0.9% exclude from analysis due to suspected allergic reaction (no data if related to EGCG)	Good safety profile of EGCG
	Placebo (saline)	3.7% related to treatment	Severe: 0% related to the treatment		-	0%	
Zhao et al., 2016 (NCT01481818) [26,27]	EGCG	Acute skin redness extending outside the radiation field was observed immediately after EGCG administration in 4.2% of patients (140 μmol/L EGCG) considered to be associated with the EGCG.	-	No other reported acute toxicity was considered to be associated with EGCG.	0%	0%	The EGCG solution was well tolerated. Maximal tolerated dose of EGCG was not found. The dose escalation stopped at 660 μmol/L.
Zhu et al., 2016/2015 (part of NCT01481818) [27,28,29]	EGCG	-	-	No reported acute toxicity was associated with EGCG.	-	0%	-
Zhu et al., 2023 [30]	Not assessed						
Patrizi et al., 2016 [31]	Cream (with EGCG, vitamin E and grape seed procyanidins	7 patients	Serious: 0%	Irritant contact dermatitis with AD flare n = 1, AD worsening n = 3, flu-like syndrome n = 1, impetigo n = 2	-	-	Good tolerance of both products
	Placebo (cream without EGCG, vitamin E and grape seed procyanidins)	4 patients	Serious: 0%	Irritant contact dermatitis n = 1, AD flare n = 2, impetigo n = 1	-	-	
Kim et al., 2014 [32]	shampoo (Rosa centifolia petals, EGCG, zinc pyrithione, climbazole	-	-	Irritation did not differ significantly between the three groups (*p* = 0.63). Of the 11 patients who complained of irritation, 9 reported pruritus, and 4 reported erythema, which are mild symptoms commonly present in patients with seborrheic dermatitis	-	-	-
	ketoconazole shampoo	-	-		-	-	-
	zinc-pyrithione shampoo	-	-		-	-	-
Kim et al., 2019 [33]	shampoo (Rosa centifolia petals, EGCG, zinc pyrithione, climbazole)	12% (discomfort)	Serious: 0%	Discomfort, including mild pruritus and burning sensation, after applying the shampoo, one patient with allergic contact dermatitis	-	0%	-
	ciclopirox olamine shampoo	30% (discomfort)	Serious: 0%	Discomfort, including mild pruritus and burning sensation, after applying the shampoo	-	4%	-

AD—atopic dermatitis; AEs—adverse events; EGCG—epigallocatechin gallate.

## Data Availability

The original contributions presented in this study are included in the article. Further inquiries can be directed to the corresponding author.

## Supplementary Materials

The following supporting information can be downloaded at: https://www.mdpi.com/article/10.3390/biomedicines13061458/s1, Figure S1: Risk of bias assessment of randomized controlled trials using RoB 2.0 tool; Table S1: Results of PubMed search. Last search: 19.03.2025, Table S2: Results of Cochrane Library search. Last search: 19.03.2025; Table S3: Results of EMBASE search. Last search: 19.03.2025; Table S4: Results of clinicaltrials.gov search. Last search: 19.03.2025; Table S5: Baseline characteristics of patients from trials included in the systematic review; Table S6: Assessment of prospective single-arm studies according to NICE criteria.
