# Supplementary material for: Efficacy and Safety of Epigallocatechin Gallate in the Treatment and Prevention of Dermatitis: A Systematic Review
Source: Biomedicines. 2025 Jun 13;13(6):1458. doi: 10.3390/biomedicines13061458 (PMC12191366; doi:10.3390/biomedicines13061458)
Supplement: Supplementary file 1 [file biomedicines-13-01458-s001.zip › biomedicines-3663256-supplementary.pdf]

Supplementary Materials

Supplementary Table S1. Results of PubMed search. Last search: 19.03.2025

Number	Keywords	Search Results
**#1**	"Dermatitis"[Mesh]	120 3362
**#2**	dermatitis OR atopic dermatitis OR Eczema OR "skin inflammation" OR Eczema OR Intertrigo OR Neurodermatitis OR Radiodermatitis	157 666
**#3**	*#1 OR #2*	157 666
**#4**	Epigallocatechin OR epigallocatechin gallate OR EGCG OR epigallo-catechin gallate OR epigallocatechin-3-gallate OR epigallocatechin-3-O-gallate	11 193
**#5**	*#3 AND #4*	44
**#6**	*#5^*	44

^ languages*: English.*

Supplementary Table S2. Results of Cochrane Library search. Last search: 19.03.2025

Number	Keywords	Search Results
**#1**	MeSH descriptor: [Dermatitis] explode all trees	5 798
**#2**	dermatitis OR atopic dermatitis OR Eczema OR "skin inflammation" OR Eczema OR Intertrigo OR Neurodermatitis OR Radiodermatitis	14 781
**#3**	*#1 OR #2*	15 442
**#4**	(Epigallocatechin OR epigallocatechin gallate OR EGCG OR epigallo catechin gallate OR epigallocatechin 3 gallate OR epigallocatechin 3 O gallate)	554
**#5**	*#3 AND #4*	13

Supplementary Table S3. Results of EMBASE search. Last search: 19.03.2025

Number	Keywords	Search Results
**#1**	'epigallocatechin gallate'/exp	16 193
**#2**	epigallocatechin OR (epigallocatechin AND gallate) OR egcg OR ('epigallo catechin' AND gallate) OR 'epigallocatechin 3 gallate' OR ('epigallocatechin 3 o' AND gallate)	19 774
**#3**	#1 OR #2	19 774
**#4**	'dermatitis'/exp OR dermatitis OR 'atopic dermatitis'/exp OR 'atopic dermatitis' OR (atopic AND ('dermatitis'/exp OR dermatitis)) OR 'skin inflammation'/exp OR 'skin inflammation' OR 'eczema'/exp OR eczema OR 'intertrigo'/exp OR intertrigo OR 'neurodermatitis'/exp OR neurodermatitis OR 'radiodermatitis'/exp OR radiodermatitis	281 225
**#5**	#3 AND #4	192
**#6**	#5*	55
**#7**	#6^	52

**filters: Humans, AND [embase]/lim NOT [medline]/lim;* ^ languages*: English.*

Supplementary Table S4. Results of clinicaltrials.gov search. Last search: 19.03.2025

Number	Keywords	Search Results
**#1**	(dermatitis OR atopic dermatitis OR Eczema OR "skin inflammation" OR Eczema OR Intertrigo OR Neurodermatitis OR Radiodermatitis) AND (epigallocatechin gallate OR EGCG)	2

Supplementary Table S5. Baseline characteristics of patients from trials included in the systematic review

Trial	Trial arms	Type of dermatitis	N	Age, mean (±SD) [years]	Sex, % of female	Baseline severity of dermatitis	Tumor type (in studies with radiation-induced dermatitis)	Treatment used before EGCG (in studies with radiation-induced dermatitis)	Dose of radiotherapy (in studies with radiation-induced dermatitis)	Performance status in ECOG scale, % (in studies with radiation-induced dermatitis)	Localization of the lesion
**Xie et al. 2023 [22] (NCT02580279)**	EGCG	Radiation-induced grade III dermatitis (treatment)	19 analyzed	53 (10.849)	53.63%	Grade III according to RTOG	Breast cancer: 42.11%<br>Lung cancer: 21.05%<br>Esophageal cancer: 36.84%	RT+CT: 68.42%<br>RT+CT+ET: 31.58%	The median dose of grade III dermatitis first observation was 44 Gy (30.6–52 Gy).<br><br>50 Gy in 25 fractions: 42.1%,<br>44 Gy in 22 fractions: 5.26%<br>59.4 Gy in 33 fractions: 31.58<br>60 Gy in 30 fractions: 15.79<br>47.88 Gy in 18 fractions: 5.26%	0: 36.84%<br>1: 63.16%<br>2: 0%	On RT site
**Zhao et al. 2022/ Zhu et al. 2020 [23]-[25] (NCT02580279)**	EGCG	Radiation-induced dermatitis (prevention)	111	46.5 (8.1)	100%	No; EGCG used as prevention of dermatitis	Breast cancer: 100%	RT: 100%<br>Adjuvant systemic therapy during study:<br>- none: 33.3%<br>- tamoxifen: 46.8%<br>- trastuzumab: 19.8%<br>- CT: 0%	50 Gy in 25 fractions: 82.9%<br>57.5 Gy in 25 fractions: 4.5%<br>60 Gy in 30 fractions: 12.6%	0: 29.7%<br>1: 70.3%<br>2: 0%	On RT site
	Placebo (saline)		54	48.4 (9.5)	100%		Breast cancer: 100%	RT: 100%<br>Adjuvant systemic therapy during study:<br>- none: 42.6%<br>- tamoxifen: 46.3%<br>- trastuzumab: 11.1%<br>- CT: 0%	50 Gy in 25 fractions: 83.3%<br>57.5 Gy in 25 fractions: 5.6%<br>60 Gy in 30 fractions: 11.1%	0: 33.3%<br>1: 66.7%<br>2: 0%	
**Zhao et al. 2016 (NCT01481818) [26]-[27]**	EGCG	Radiation-induced grade I dermatitis (treatment)	24	Median (range): 44 (22-63)	100%	Grade I according to RTOG	Breast cancer: 100%	RT: 100%	50 Gy in 25 fractions over 5 weeks	0: 41.7%<br>1: 58.3%<br>2: 0%	On RT site
**Zhu et al. 2016/2015 (part of NCT01481818) [28]-[29], [27]**	EGCG	Radiation-induced grade I dermatitis (treatment)	49	Median (range): 45 (22-64)	100%	Grade I according to RTOG	Breast cancer: 100%	RT: 100%	RT was fractioned in 2 Gy five days a week up to 50 Gy	0: 46.9%<br>1: 53.1%<br>2: 0%	On RT site
**Zhu et al. 2023 [30]**	EGCG	Radiation-induced dermatitis (prevention)	43	Median (range): 45 (26-67)	100%	No; EGCG used as prevention of dermatitis	Breast cancer: 100%	RT: 100%	-	-	On RT site
	No EGCG		43		100%		Breast cancer: 100%	RT: 100%	-	-	On RT site

Trial	Trial arms	Type of dermatitis	N	Age, mean (±SD) [years]	Sex, % of female	Baseline severity of dermatitis	Tumor type (in studies with radiation-induced dermatitis)	Treatment used before EGCG (in studies with radiation-induced dermatitis)	Dose of radiotherapy (in studies with radiation-induced dermatitis)	Performance status in ECOG scale, % (in studies with radiation-induced dermatitis)	Localization of the lesion
**Patrizi et al. 2016 [31]**	Cream with EGCG, vitamin E and grape seed procyanidins)	Mild-to-moderate atopic dermatitis	20 included in analysis	27 (range: 6-69) Patients ≥18 years: 65%	70%	Mild-to-moderate atopic dermatitis (IGA 2 or 3)	-	-	-	-	Face and/or neck
	Placebo (cream without EGCG, vitamin E and grape seed procyanidins)		19 included in analysis	18.4 (range: 6-60) Patients ≥18 years: 53%	63.2%	-	-	-	-	-	
**Kim et al. 2014 [32]**	shampoo (Rosa centifolia petals, EGCG, zinc pyrithione, climbazole)	Scalp seborrheic dermatitis with clinical severity score of ≥3	25	38.4 (8.7)	80%	Clinical severity score (mean, SD): 4.2 (1.0)	-	--	-	-	scalp
	ketoconazole shampoo		25	37.7 (9.2)	72%	Clinical severity score (mean, SD): 4.3 (1.2)	-	-	-	-	
	zinc-pyrithione shampoo		25	36.0 (9.0)	56%	Clinical severity score (mean, SD): 4.1 (1.1)	-	-	-	-	
**Kim et al. 2019 [33]**	shampoo (Rosa centifolia petals, EGCG, zinc pyrithione, climbazole)	Scalp seborrheic dermatitis with clinical severity score of >3	25	36.2 (8.7)	60%	Clinical severity score (mean, SD): 4.40 (1.08)	-	-	-	-	scalp
	ciclopirox olamine shampoo		23	34.6 (8.2)	70%	Clinical severity score (mean, SD): 4.47 (1.61)	-	-	-	-	

RT - radiation therapy, CT chemo therapy, ET endocrine therapy.SD – standard deviation.

Supplementary Table S6. Assessment of prospective single-arm studies according to NICE criteria.

Criterium	Xie et al. 2023 [22]	Zhao et al. 2016 (NCT01481818) [26]-[27]	Zhu et al. 2016/2015 (part of NCT01481818) [28]-[29], [27]
**Case series collected in more than one center, i.e., multi-center study**	YES (1 point)	NO (0 points)	NO (0 points)
**Is the hypothesis/aim/objective of the study clearly described?**	YES (1 point)	YES (1 point)	YES (1 point)
**Are the inclusion/exclusion criteria (case definition) clearly reported?**	YES (1 point)	YES (1 point)	YES (1 point)
**Is there a clear definition of the outcomes reported?**	YES (1 point)	YES (1 point)	YES (1 point)
**Were data collected prospectively?**	YES (1 point)	YES (1 point)	YES (1 point)
**Is there an explicit statement that patients were recruited consecutively?**	NO (0 points)	NO (0 points)	NO (0 points)
**Were the main results/findings of the study clearly described?**	YES (1 point)	YES (1 point)	YES (1 point)
**Were the analyzed endpoints assessed in strata (groups of patients distinguished by, for example, the stage of disease advancement, abnormal test results, patient characteristics)??**	NO (0 points)	YES (1 point)	NO (0 points))
**Sum of points:**	**6 points**	**7 points**	**6 points**

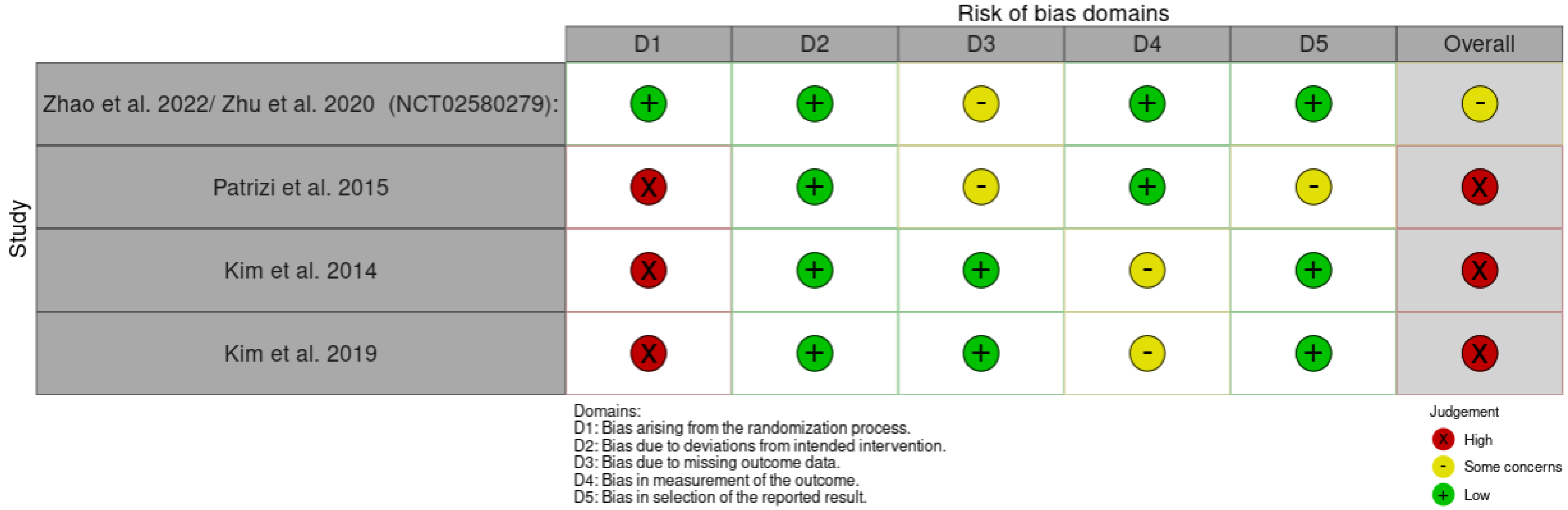

Supplementary Figure S1. Risk of bias assessment of randomized controlled trials using RoB 2.0 tool.

Trials: Zhao et al. 2022/Zhu et al. 2020 (NCT02580279) [23–25], Patrizi et al. 2015 [31], Kim et al. 2014 [32], and Kim et al. 2019 [33].
